# Cued reactivation during slow-wave sleep induces brain connectivity changes related to memory stabilization

**DOI:** 10.1038/s41598-018-35287-6

**Published:** 2018-11-16

**Authors:** Ruud M. W. J. Berkers, Matthias Ekman, Eelco. V. van Dongen, Atsuko Takashima, Markus Barth, Ken. A. Paller, Guillén Fernández

**Affiliations:** 1 Donders Institute for Brain, Cognition and Behaviour, Radboud University Medical Center, Nijmegen, The Netherlands; 2 Max Planck Institute for Human Cognitive & Brain Sciences, Leipzig, Germany; 3 Donders Institute for Brain, Cognition and Behaviour, Radboud University, Nijmegen, The Netherlands; 4 Max Planck Institute for Psycholinguistics, Nijmegen, The Netherlands; 5 Centre for Advanced Imaging, The University of Queensland, Brisbane, Australia; 6 Northwestern University, Evanston, Illinois, USA

## Abstract

Memory reprocessing following acquisition enhances memory consolidation. Specifically, neural activity during encoding is thought to be ‘replayed’ during subsequent slow-wave sleep. Such memory replay is thought to contribute to the functional reorganization of neural memory traces. In particular, memory replay may facilitate the exchange of information across brain regions by inducing a reconfiguration of connectivity across the brain. Memory reactivation can be induced by external cues through a procedure known as “targeted memory reactivation”. Here, we analysed data from a published study with auditory cues used to reactivate visual object-location memories during slow-wave sleep. We characterized effects of memory reactivation on brain network connectivity using graph-theory. We found that cue presentation during slow-wave sleep increased global network integration of occipital cortex, a visual region that was also active during retrieval of object locations. Although cueing did not have an overall beneficial effect on the retention of cued versus uncued associations, individual differences in overnight memory stabilization were related to enhanced network integration of occipital cortex. Furthermore, occipital cortex displayed enhanced connectivity with mnemonic regions, namely the hippocampus, parahippocampal gyrus, thalamus and medial prefrontal cortex during cue sound presentation. Together, these results suggest a neural mechanism where cue-induced replay during sleep increases integration of task-relevant perceptual regions with mnemonic regions. This cross-regional integration may be instrumental for the consolidation and long-term storage of enduring memories.

## Introduction

The neural representations of recently acquired declarative memories are thought to be selectively reactivated during ensuing rest periods, particularly during slow-wave sleep^1,2^. For instance, hippocampal place cells in rats that co-activated during active behavior were found to be reactivated in concert during ensuing slow-wave sleep^3^. Later studies found that this coordinated reactivation, also dubbed replay, extended to the perceptual cortex involved in the initial processing of the stimulus, such as the visual cortex^4^. Furthermore, evidence of neural replay has also been found in the medial prefrontal cortex^5–7^. These results demonstrate the occurrence of neural memory replay in rodents, that is behaviorally relevant for the retention of associative memories^8^.

Neural memory reactivation is difficult to observe in humans, as its timing is generally unknown. However, reactivation can also be induced using cues associated with information learned previously, termed “targeted memory reactivation”. For example, presenting an odor during slow-wave sleep induces activation of the hippocampus and stabilizes associated memory traces^9^. Furthermore, whereas studies have found that audio-visual-spatial associations can be stabilized in a targeted manner using specific auditory cues^10,11^, these cueing effects can also affect all associations acquired within the same learning context^12^. Van Dongen and colleagues^13^ performed targeted memory reactivation in the magnetic resonance (MR) scanner using auditory cues that were paired with specific object-location associations. Even though no consistent effect of cueing on memory stabilization was found, individual differences in the effect of cueing positively correlated with activity in the hippocampus, thalamus and cerebellum during slow-wave sleep. Given repeated earlier findings of memory improvement due to auditory cues presented during slow-wave sleep^12,14,15^, it is likely that the noisy scanner environment contributed to excessive variability here, with some individuals effectively tuning out all auditory input, or alternatively that cueing affected all associations acquired within the same learning context^13^.

Here, we re-analyzed data acquired by van Dongen and colleagues^13^ using a principled graph theoretical analysis approach to characterize whole-brain connectivity changes. Triggering reactivation of memory traces with auditory cues could induce a process akin to spontaneous memory replay, which likely involves whole-brain connectivity changes facilitated by synchronous brain states during slow-wave sleep^16,17^. In this study, we assess whether those effects are reflected in shifts in functional connectivity patterns in response to exogenous (cued) memory reactivation that may be related to systems consolidation. Conventional analysis methods are often unable to capture the coordinated whole-brain connectivity changes expected during memory replay. Thus, a network perspective can be leveraged using methods developed within the framework of graph theory^18^. The *participation coefficient* is a metric that captures the extent to which a region integrates and distributes information as a result of the number and positioning of their contacts in the network^19^. Specifically, it quantifies network integration by looking at the importance of a given node for interactions between subnetworks, by measuring the connectivity of a region within a module compared to the connectivity to other modules. The participation coefficient can, as such, be used to index the integration of a given voxel (node) in the wider brain (network) during cued reactivation of visuospatial associations during slow-wave sleep.

If a cue induces memory reactivation akin to replay, and this indeed plays a role in memory consolidation involving a hippocampal-neocortical trace shift, then one should expect a coordinated neural activation of multiple regions, including the sensory features of the cued memory (in this case visual object features in the occipital cortex^20^ and the spatial layout in the parahippocampal gyrus^21^) as well as key mnemonic regions (such as the hippocampus, thalamus, and medial prefrontal cortex). This increased integration of these and other regions would be expected to be related to memory stabilization and an increased involvement of the neocortex in the memory trace across sleep.

## Methods

The Materials and Methods have been described extensively in the original report^13^. Here, we summarize relevant details about the experimental setup, supplemented with details about the data analysis performed for this report.

### Participants

This re-analysis includes data from all 22 participants included in the original report (see for complete description). Briefly, 56 participants were initially recruited from the online research participant system of the Radboud University Nijmegen (age range 18–27; 14 males). Twenty-two participants in the sample reached a sufficient amount of slow-wave sleep stage in the scanner to allow at least 80% of the cueing protocol to be completed whilst in this stage. Furthermore, these participants did not display micro-arousals in response to sound presentations, nor did they report explicit knowledge of the sounds having been presented while they slept. The experiment was conducted in accordance with national legislation for the protection of human volunteers in nonclinical research settings and the Helsinki Declaration and approved by the local ethics committee (CMO Arnhem-Nijmegen, Radboud University Medical Center). Participants provided written informed consent prior to participation and were compensated for participation with course credits or a monetary fee.

### Procedures

**Figure 1:**
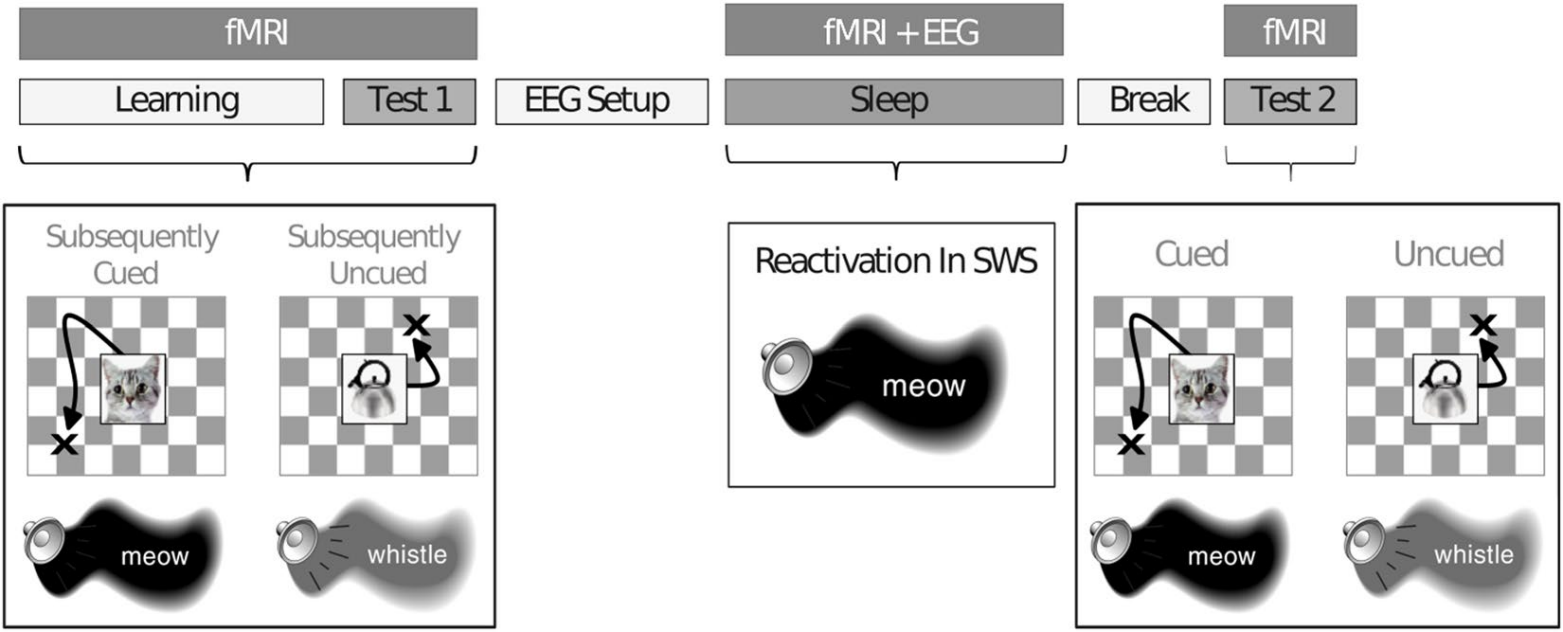
Schematic depiction of the experimental procedure. Participants learned 50 object-location associations, presented simultaneously with object-related sounds, inside the MR-scanner. After a baseline test (Test 1), participants were set up for polysomnographic recordings and went to sleep inside the MR-scanner. Half of the learned associations were cued with sounds during slow-wave sleep. After awaking and a short break, participants performed another post-sleep test (Test 2). Reprinted from^13^.

The experiment started between 7 and 8 PM, and participants went to sleep between 10 PM and midnight (see Fig. 1). Participants were placed in the MR scanner, and the sound volume was individually calibrated to a level at which the participant could distinguish individual sounds while the scanner was operating. Participants learned 50 object-location associations, each object-location pair being presented together with a unique sound associated with the object, inside the scanner. When all associations were learned to criterion (all objects placed within 4 cm from the correct location on two subsequent rounds), a pre-test was performed on all associations (Test 1). Next, participants were prepared outside the scanner for polysomnographic recordings, and placed in the scanner again for a 2 hr rest/sleep period, while electroencephalograph (EEG) was monitored online and fMRI data was continuously acquired. When participants had entered a stable slow-wave state for a period of time as visible on the ongoing polysomnography^22^, sounds were presented to the participants using an MR-compatible headphone. Specifically, half of the sounds that were associated with specific object-location associations during learning were presented as ‘cue sounds’ during sleep (25 sounds presented twice, for a total of 50 cue sound presentations). Moreover, control sounds that had not been previously associated with learning materials were presented (5 sounds presented five times, for a total of 25 control sound presentations). Cue and control sounds were grouped in blocks of five 500 ms presentations, each separated by a 4–8 s jittered inter-stimulus interval (total block duration: 32 seconds). The total amount of trial presentations was limited due to inherent practical difficulties with obtaining sufficient periods of slow-wave sleep to play the sound recordings. Therefore, with this limitation in mind, a 2:1 ratio of cue trials versus control trials was implemented to maximize the likelihood to find an effect of reactivation on memory stabilization. The order of cue sound and control sound presentations was randomized in order to prevent temporal order confounds. After a 2 hr sleep, participants were awoken and subsequently were tested on all object-location associations (Test 2).

### Object-location test

The object-location task was performed in three stages: learning, Test 1 and Test 2. Here, we focus on the behavioral data from Test 1 and Test 2 for further analysis. Participants learned the location of 50 object pictures. For each participant, 50 objects were randomly assigned to 50 screen locations (screen size: 47 × 35 cm, resolution: 1024 × 768, viewing distance: 60 cm). In the learning phase, participants first passively viewed all 50 objects in their respective screen locations (duration: 3 s presentation followed by a 1 s inter-stimulus interval), paired with hearing the object-related sound (e.g. a cat’s ‘meow’ when the object was a cat, sound duration: 500 ms). Next, the learning phase continued with several iterative rounds of active learning, where an object was presented at the center of the screen simultaneously with the auditory stimulus. Participants were then required to place the object in its original location using an MR-compatible joystick, and to press a button to confirm the object placement (self-paced timing). Feedback was given for each trial by displaying the object in the correct location for 3 s after a response was made. Objects were presented in a random order, and with every new round those objects that had been placed within 4 cm from the correct location on the two preceding rounds were excluded. Upon reaching criterion performance on all objects, participants performed the pre-sleep test (Test 1) for all objects without feedback. An identical post-sleep test was performed after sleep (Test 2). During Test 2, half of the object-location associations had previously been cued during sleep (‘cued associations’), whereas the other half of the associations had not been previously cued (‘uncued associations’). During tests, object placement was followed by an inter-stimulus period of fixation (duration jittered between 3 and 5 s).

### MRI Data Acquisition

Participants were scanned using a reduced-noise Echo-Planar Imaging (EPI) sequence with sinusoidal gradients to avoid acoustic resonances of the scanner^23^. Functional (T2*) images were acquired with whole-brain coverage (28 axial slices, ascending slice acquisition, repetition time (TR) = 2,511 ms, echo time (TE) = 38 ms, 90° flip angle, matrix = 64 × 64, bandwidth = 1,502 Hz per voxel, slice thickness = 3.5 mm, slice gap = 15%, field of view (FOV) = 244 mm). Structural (T1) images were acquired with a magnetization-prepared rapid acquisition gradient echo sequence (176 sagittal slices, TR = 2,250 ms, TE = 2.95 ms, 15° flip angle, matrix = 256 × 256, slice thickness = 1.0 mm, FOV = 256 mm).

### MRI Data Preprocessing

The fMRI data acquired during Test 1, the sleeping period and Test 2 were preprocessed using standard routines implemented in SPM8 (www.fil.ion.ucl.ac.uk). The first five volumes of each functional EPI run were discarded, and an outlier algorithm was used to check for corrupted slices from each image and replaced using between-volume interpolation. The functional images were then realigned, and coregistered to the structural image, spatially normalized to the Montreal Neurological Institute (MNI) EPI template (resampled at voxel size 2 × 2 × 2 mm), and smoothed using a Gaussian kernel (8 mm full-width at half maximum, FWHM). The structural images were segmented into gray matter, white matter, cerebrospinal fluid, and residual compartments (outside brain and the skull) using the unified segmentation algorithm as implemented in SPM8. These compartments were used as masks to extract the mean intensity level across the whole time-series, and entered as compartment regressors to account for effects related to non-specific signal fluctuations.

### Network analysis during cueing

We employed a graph theoretic framework^18,24^ to analyze connectivity patterns during the presentation of sound cues in stable periods of slow-wave sleep. The aim was to measure dynamic changes in the whole-brain network properties during memory reactivation (by contrasting the presentation of cue sounds versus control sounds), and specifically changes in integration of certain brain regions with the whole brain network. Task-related connectivity was estimated using the common beta time-series approach^25^. Beta time-series are obtained by convolving each trial with an HRF separately (a separate regressor is included for each trial) and fitting the BOLD time-series using a standard GLM using FSL’s Feat (including voxel-wise pre-whitening and AR(1) estimation). Resulting trial-specific beta estimates are then concatenated over trials to form a beta time-series. These beta time-series as such represent task-specific signal fluctuations.

A high-pass filter with a cut-off period of 128 s was applied to the functional time series. Sound presentation during sleep was modelled using a separate regressor for each sound presentation (to obtain trial-specific beta-estimates for the 50 cue sound presentations and 25 control sound presentations, respectively) with a duration of 5 seconds each. We used a duration of 5 seconds to model cue presentation (which lasted 500 ms itself) to account for timing uncertainties with regard to the onset and duration of the induced reactivation of the memory trace in response to the cue. First, the induction of memory reactivation by auditory input might depend on specific spindle-ripple events occurring during cortical up-states^16,17,26^. It is therefore difficult to be very precise regarding a specific time-point where the auditory signal could potentially induce memory reactivation. Furthermore, once the auditory stimulus has reached the cortex and has induced reactivation of the memory trace, it is unclear how long the induced memory reactivation could last. To deal with this uncertainty, we modelled cue presentation with an extended boxcar-window, rather than a stick function that would restrict us to a specific time-point. The design matrix also included six regressors of no interest to account for head movement, and the three compartment regressors to account for non-specific signal fluctuations. The resulting beta estimates from the GLM were concatenated separately for cue sound and control sound trials. This procedure resulted in two separate beta time-series (i.e., consisting of 50 data points for cue sounds and 25 data points for control sounds) for all voxels in the brain. Next, voxel-wise correlation coefficients of beta time-series were computed to quantify pairwise functional connectivity for each condition separately (i.e., cue sounds and control sounds). These voxel-wise connectivity matrices were thresholded by preserving only significant connections (*p* < 0.05 False discovery rate (FDR) corrected) and setting all other connections, as well as all negative correlations to zero.

The large range of graph theoretic parameters that can be measured can result in many degrees of freedom in the analysis. Here, we restricted the analysis a priori to one metric of interest, namely the participation coefficient^19,27,28^. The participation coefficient is argued to be the most appropriate measure of hubness, and quantifies for each node (i.e. voxel) the diversity of its inter-modular connections^29^, specified as the amount of connections with nodes in other modules relative to the total amount of connections. It therefore is a measure of the importance of a given node for global inter-modular integration across the brain.

Following the two-step procedure used by Power and co-workers^19^, modular network structure was derived based on a coarse connectivity matrix, using nodes based on 115 anatomical regions defined by the AAL (Automated Anatomical Labeling) atlas^28,30^. Beta time-series were averaged across voxels within an anatomical region, and a 115 × 115 region-by-region connectivity matrix was constructed. After thresholding this connectivity matrix (edges > 0, *p* < 0.05 FDR corrected), the matrix was parcellated into subnetworks using modularity detection according to the Louvain method^31^. Of note, the AAL-based connectivity matrix was only used to estimate the global community structure using community detection algorithms. This procedure is computationally intensive at the voxel level, but is computationally tractable using a down-sampled dataset on the basis of an anatomical parcellation^19,29^.

Using the community structure estimated at the AAL-level, the participation-coefficient itself was then calculated again at the voxel-level, thereby retaining the fine-grained specificity at this step of analysis. Specifically, each voxel in the whole-brain voxel-wise connectivity matrix was assigned to a module, allowing the calculation of their participation coefficient ranging from 0 (provincial hub: connections are only present within its module) to 1 (connector hub: connections are only present with other modules). The resulting participation coefficient images for cue versus control sounds were compared across subjects using an undirected paired t-test.

### Seed-based connectivity analysis during cueing

The previous analysis determined which region increased in global inter-modular network integration. We performed a follow-up seed-based functional connectivity analysis to determine which parts of the brain were more connected with the region found during the presentation of cue versus control sounds. Therefore, we correlated the average beta time-series across all voxels of the seed region found in the network analysis (cluster-forming threshold at *Z* > 2.33, the resulting cluster was significant when correcting for multiple comparisons for the whole brain using Gaussian Random Field Theory at a threshold of *p* < 0.05) with voxels in the rest of the brain separately for cue and control beta time-series. The resulting correlation maps were subsequently contrasted. Small-volume correction was used for regions that were strongly suggested to be involved on the basis of the earlier report of this data and the literature on sleep-related memory consolidation. First, the parahippocampal gyrus is activated by the object-location retrieval task^13^, and is implicated in encoding visuo-spatial memories^32–34^. Furthermore, cortical regions that are involved in initial processing at encoding are presumed to be part of the reactivated memory trace along with the hippocampus as a connecting hub^35–37^. Second, the thalamus and hippocampus display cue-selective activity related to overnight memory stabilization^13^ and have both been implicated in sleep-related memory reprocessing and replay-related spindle-ripple events during slow-wave sleep that facilitates information transfer^16,17,38,39^. Anatomical masks as implemented in the IBASPM 71 Atlas of the WFU Pick Atlas Tool in SPM were used for small-volume correction^40^. For statistical inferences, cluster-forming thresholds at *Z* > 2.33 were used, and the resulting clusters were tested correcting for multiple comparisons for the whole brain using Gaussian Random Field Theory. Next, multiple-comparisons correction on anatomical regions of interests was performed on the test-statistics obtained from small-volume analyses. The false-discovery rate was controlled using the Benjamin & Hochberg procedure^41^ to calculate the critical p-value (*p* = 0.031 for this contrast) for all regions of interest (ROIs).

### Activity analysis during pre- and post-sleep test

We next assessed whether network integration of visual cortex during cueing in slow-wave sleep results in increased involvement of cortical associative regions at retrieval. The parahippocampal gyrus forms a candidate region for increased involvement with consolidation, as it is activated by the object-retrieval task, displays increased connectivity with the early visual cortex during cueing, and is generally implicated in processing visuospatial memories^32–34,42^. The parahippocampal gyrus is presumed to be the cortical memory hub that would show greater involvement with greater systems consolidation, spurred by cue-induced network participation of the early visual cortex. For this reason, we contrasted the neural activations observed during the pre-sleep (Test 1) and post-sleep (Test 2) tests, and related this contrast to the participation coefficient of the occipital cortex as observed in the graph theory analysis (see Results section). The fMRI data for Test 1 and Test 2 were analyzed in one GLM using a multi-session design in SPM. This model included regressors for cued and uncued associations, which were modelled as delta functions to be convolved with the canonical hemodynamic response function (HRF), along with temporal derivatives provided by SPM8. The design matrix included six regressors of no interest per test session to account for head movement using the realignment parameters, as well as compartment regressors accounting for non-specific signal fluctuations. Furthermore, a high-pass filter with a cutoff period of 128 s was implemented to remove low-frequency fluctuations from the time series. Parameter images were generated on the first level (for each individual participant) based on a contrast between all trials of Test 1 and all trials of Test 2, to assess changes in retrieval activity from the pre- to the post-sleep test. We collapsed the brain response to cued associations and uncued associations because increased network participation of the occipital cortex in response to the presentation of cue sounds was not specifically related to only the cued associations in the learned set (see ‘Results’). These contrast images were entered into a second-level GLM with one covariate, namely the participation coefficient of the occipital cortex, as found during cue sound (sounds that were associated with previously learned associations) versus control sound (the sounds that had no association to previously learned associations) presentation in the sleep phase. Small-volume correction was used for the bilateral parahippocampal gyri. Here, an anatomical mask of the parahippocampal gyrus implemented in the IBASPM 71 Atlas of the WFU Pick Atlas Tool in SPM was used for small-volume correction^40^. For statistical inferences, cluster-forming thresholds at *Z* > 2.33 were used, and the resulting clusters were tested correcting for multiple comparisons for the whole brain using Gaussian Random Field Theory. Multiple-comparisons correction on anatomical regions of interests was performed on the test-statistics obtained from small-volume analyses. The false-discovery rate was controlled using the Benjamin & Hochberg procedure^41^ to calculate the critical *p*-value (*p* = 0.042 for this analysis) for each ROI.

## Results

### Network changes following cueing

**Figure 2:**
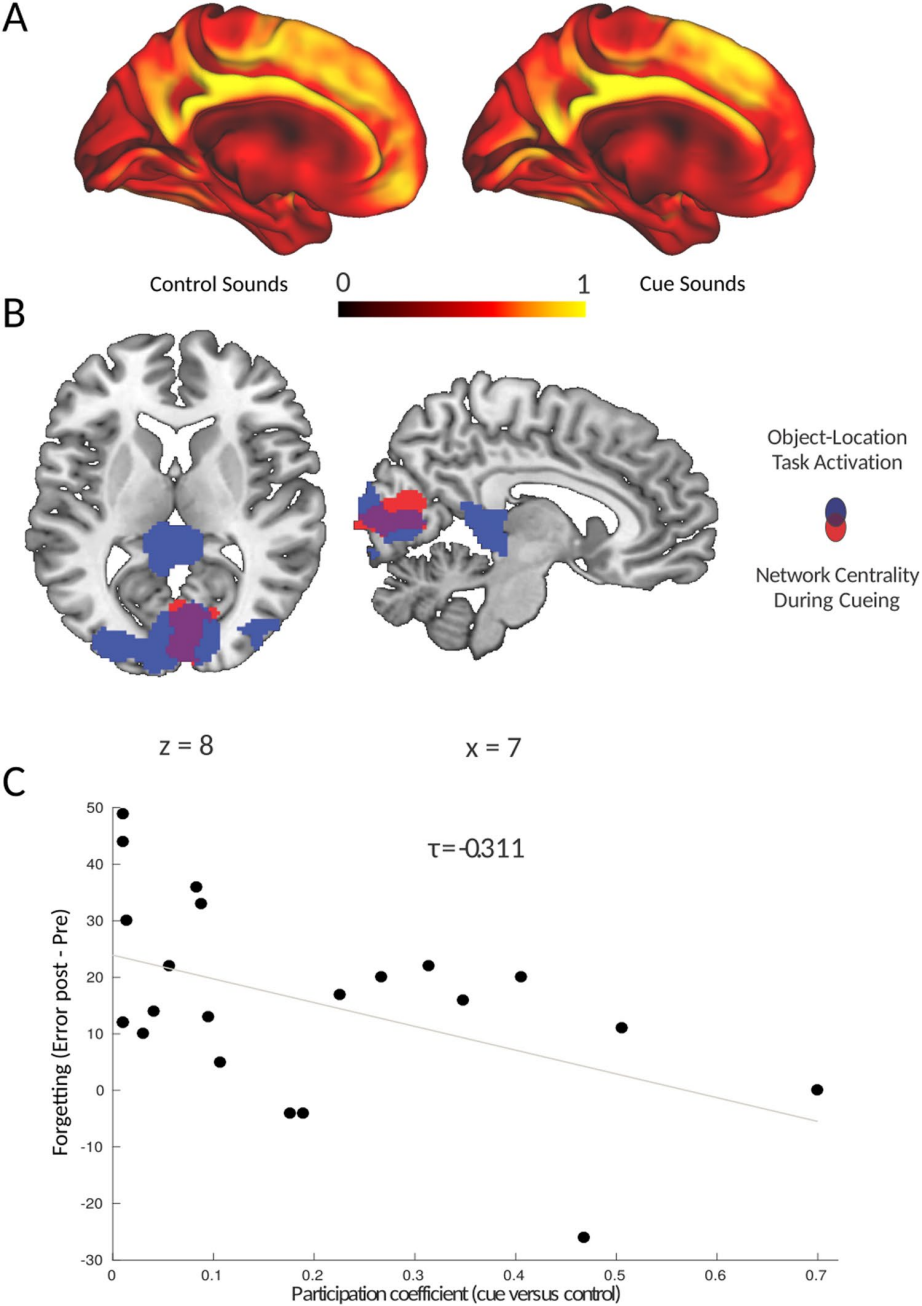
Reactivation-related changes in network integration predict memory stabilization. (**A**) Participation Coefficient mapped for every voxel in the brain during the presentation of control sounds (not previously paired with object-location information, left panel) and cue sounds (previously paired with object-location associations, right panel). (**B**) Increased network integration was found only in the occipital cortex during the presentation of cue sounds versus control sounds during slow-wave sleep. This region overlapped with the set of regions activated in response to the object-location association task. Parametric maps were superimposed onto a template brain, using the cluster-forming threshold of Z > 2.33. (**C**) The increase in participation coefficient of the occipital cortex during cueing predicted memory stabilization, indicated by reduced forgetting (expressed as the difference in error distances) between test 1 (pre) and test 2 (post).

We probed changes in network dynamics in response to the presentation of cue sounds versus control sounds during slow-wave sleep. The presentation of cue sounds was hypothesized to induce reactivation of the corresponding memory trace, which would presumably be reflected in increased integration of areas that represent sensory features of the cued memory representation, e.g. occipital cortex. Indeed, we found a specific increase in the participation coefficient of early visual (occipital) cortex (peak MNI coordinates: *x, y, z* = [4, −70, 6], *z*_21_ = 3.12, cluster-forming threshold at *Z* > 2.33, corrected for the whole brain at *p* < 0.05 using Gaussian Random Field Theory), indicating that occipital cortex displays an increased inter-modular connectivity when cue sounds were presented during deep sleep (see Fig. 2A and Table 1). This region exhibited overlap with regions that were activated in response to the object-location task during Test 1 (see Fig. 2B). This overlap is suggestive of a reprocessing of the original visuo-spatial neural memory trace in response to auditory cues presented during sleep, in the absence of any visual input.

**Table 1:** Brain regions where the participation coefficient is higher during the presentation of cue sounds (previously paired with object-location associations, see right panel of Fig. 2A), versus control sounds (not previously paired with object-location information, see left panel of Fig. 2A). Listed are the local maxima of the significant cluster and the corresponding peak z-value.

#	Region	X	Y	Z	Peak Z-value	Cluster Size	P-value
1	Lingual Gyrus	4	−70	6	3.12	464	0.03**
	Cuneus	4	−82	8	3.06		
	Cuneus	4	−72	14	3.04		

**Region was significant using multiple comparison correction for the whole brain using Gaussian Random Field Theory and a threshold of p < 0.05.

**Figure 3:**
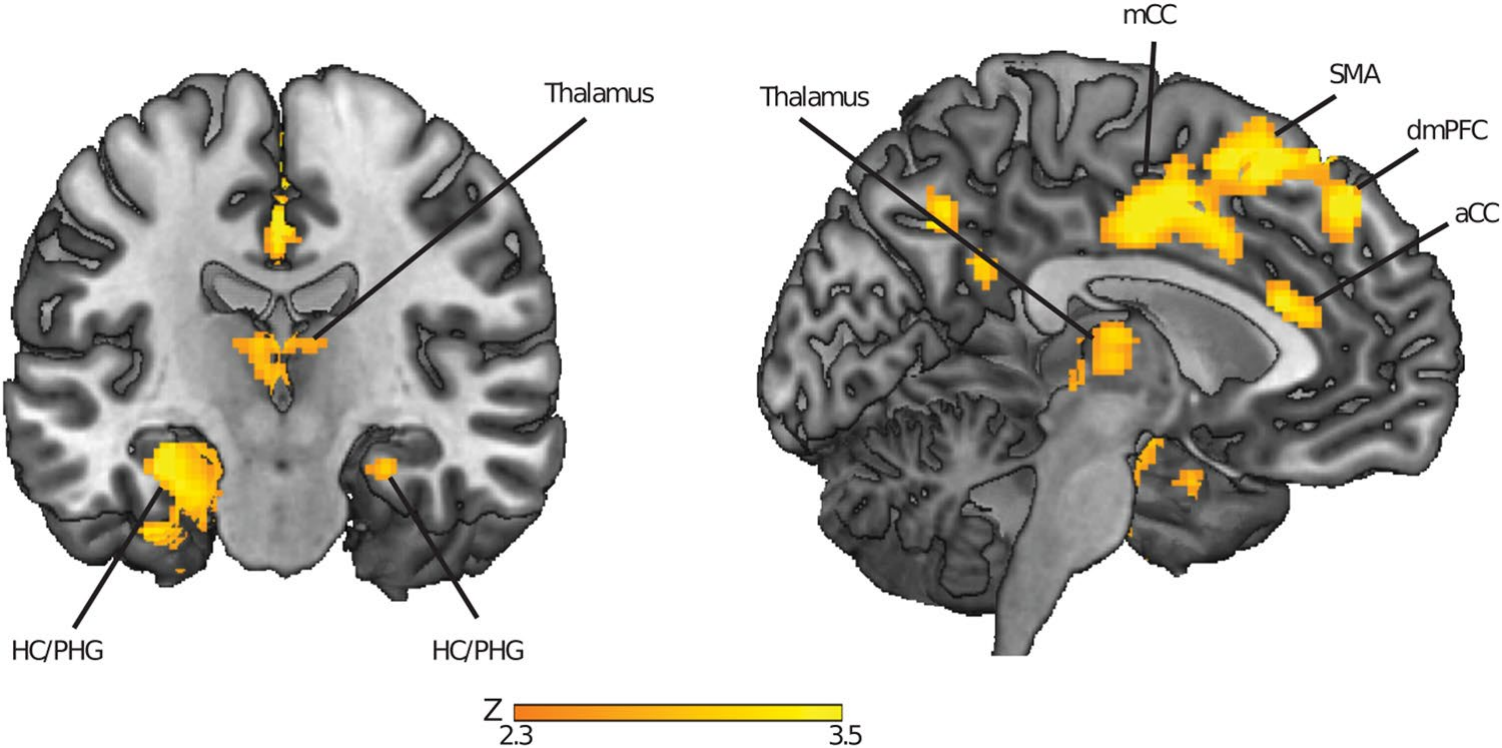
Regions displaying greater coupling with occipital cortex during cueing in slow-wave sleep. Parametric maps were superimposed onto a template brain, using the cluster-forming threshold of Z > 2.33. HC = Hippocampus, PHG = Parahippocampal Gyrus, aCC = Anterior Cingulate Cortex, mCC = Middle Cingulate Cortex, SMA = Supplementary Motor Area, dmPFC = Dorsomedial Prefrontal Cortex.

To determine what brain regions this area in the occipital cortex preferentially connects to when recruited in the network by the auditory cues, we performed a seed-based functional connectivity analysis based on the beta time-series. The seed was defined as the region in the cortex that displayed an increase in participation coefficient during cueing (cluster-forming threshold at *Z* > 2.33). The task contrast was formed by the presentation of cue sounds versus control sounds. The occipital cortex was found to be connected to the left hippocampus (peak MNI coordinates: *x, y, z* = [−32, −16, −18], *Z*_21_ = 3.36), right hippocampus (peak MNI coordinates: *x, y, z* = [22, −2, −22], *Z*_21_ = 2.98), left parahippocampal gyrus (peak MNI coordinates: *x, y, z* = [−26, −22, −26], *Z*_21_ = 3.10), left thalamus (peak MNI coordinates: *x, y, z* = [−4, −2, −0], *Z*_21_ = 3.11), right thalamus (peak MNI coordinates: *x, y, z* = [2, −16, −12], *Z*_21_ = 2.67), and a medial prefrontal region extending from premotor cortex to anterior cingulate cortex and into dorsomedial prefrontal cortex (see Fig. 3 and Table 2, all cluster-forming thresholds at *Z* > 2.33, cluster-size corrected for the whole brain, or reduced search regions based on predefined anatomical areas for the bilateral hippocampus, parahippocampal gyrus and thalamus, thresholded at *p* < 0.05 using Gaussian Random Field Theory). This increase in connectivity could not be attributed to negative correlation values in the control condition, as visible from extracted correlation values in peak regions (see Supplementary Fig. 1). Thus, the reported increases in seed-based connectivity during cueing can be attributed to an increase in positive correlation values during cueing. In sum, during cueing, the occipital cortex was connected to several regions where memory replay has been shown to occur (the hippocampus, medial prefrontal cortex), and that are involved in oscillatory patterns of activity (spindles, ripples, slow-waves) that allow for systematic interactions between brain regions (e.g., the hippocampus, medial prefrontal cortex and thalamus).

**Table 2:** Brain regions that show a significantly higher connectivity with the early visual cortex during the presentation of cue sounds, versus control sounds (not previously paired with object-location information, left panel).

#	Region	X	Y	Z	Peak Z-value	Cluster Size	P-value
1	anterior cingulate cortex	16	14	36	3.75	2218	0.003**
	dorsomedial prefrontal cortex	−10	30	54	3.65		
	dorsomedial prefrontal cortex	−10	36	56	3.5		
	rostral cingulate cortex	−2	−6	42	3.38		
	dorsomedial prefrontal cortex	−6	44	44	3.35		
	Supplementary motor area	0	2	44	3.35		
2	L hippocampus	−32	−16	−18	3.36	271	0.006*
3	R hippocampus	26	−18	−18	2.69	13	0.031*
4	L parahippocampal gyrus	−26	−22	−26	3.1	180	0.010*
5	L thalamus	−6	−16	12	2.81	215	0.013*
6	R thalamus	2	−16	12	2.67	74	0.016*

Listed are the local maxima of the significant cluster, as well as clusters found in pre-defined anatomical regions of interest. The critical p-value was calculated to correct for multiple (six) comparisons done across anatomical regions of interest, using the Benjamin & Hochberg procedure (1995) to control the false discovery rate. The critical p-value thus calculated was p = 0.031.

**Region that was significant using multiple comparison correction for the whole brain using Gaussian Random Field Theory and a threshold of p < 0.05.

*Region that was significant using a multiple comparison correction for a reduced search volume defined by anatomical regions of interest, and a subsequent correction for the false discovery rate (critical p-value = 0.031).

### Network dynamics related to behavioral cueing effect

Memory performance decreased on average (Test 1 performance: error = 2.74 ± 0.12 cm; Test 2 performance: error = 3.12 ± 0.14: Δerror = 0.37 ± 0.08; t_21_ = 4.48, p < 0.001), both for cued (Δerror = −0.44 ± 0.11 cm; *t*_21_ = 3.86, *p* = 0.001), and uncued associations (Δerror = −0.33 ± 0.11 cm; *t*_21_ = 2.56, *P* = 0.018)). There was no effect of cueing on participants’ memory accuracy as tested after sleep (*F*_1,21_ = 0.83; *p* = 0.374). Thus, there was forgetting across the sleep/rest session, and this forgetting appeared to be similar for cued and uncued object-location associations.

However, there was substantial variability in the effect of cueing on retention. Therefore, different neural responses to cueing might explain individual differences in the effect of cueing. In particular, increased network participation of occipital cortex could be an index of how much the visual-spatial memory trace became reactivated in a given subject when auditory cues were presented. Indeed, the increased participation coefficient during cue sound presentation was found to be associated (see Fig. 2C) with reduced overnight forgetting of visual-spatial object locations (τ_21_ = −0.311, *p* = 0.048). This relationship was specifically present for the visual-spatial locations that were associated with sound cues (τ_21_ = −0.338, *p* = 0.032), but not for other locations (τ_21_ = −0.211, *p* = 0.183), although the difference in these correlations did not reach significance (Williams-Hotelling test, t = 0.43, *p* = 0.668). Furthermore, the increased participation coefficient during cue sound presentation was specifically related to overnight forgetting, and not to general memory performance as assessed at Test 1 (τ_21_ = 0.048, *p* = 0.777) or Test 2 (τ_21_ = −0.092, *p* = 0.572). This result indicates that those participants who displayed increased network participation of occipital cortex experienced a reduction in overnight forgetting of all associations. This effect was not specific to the cued items, but extended to uncued object-location associations encoded within the same spatial layout and temporal learning context.

### Overnight activity differences related to network integration during cueing

**Figure 4:**
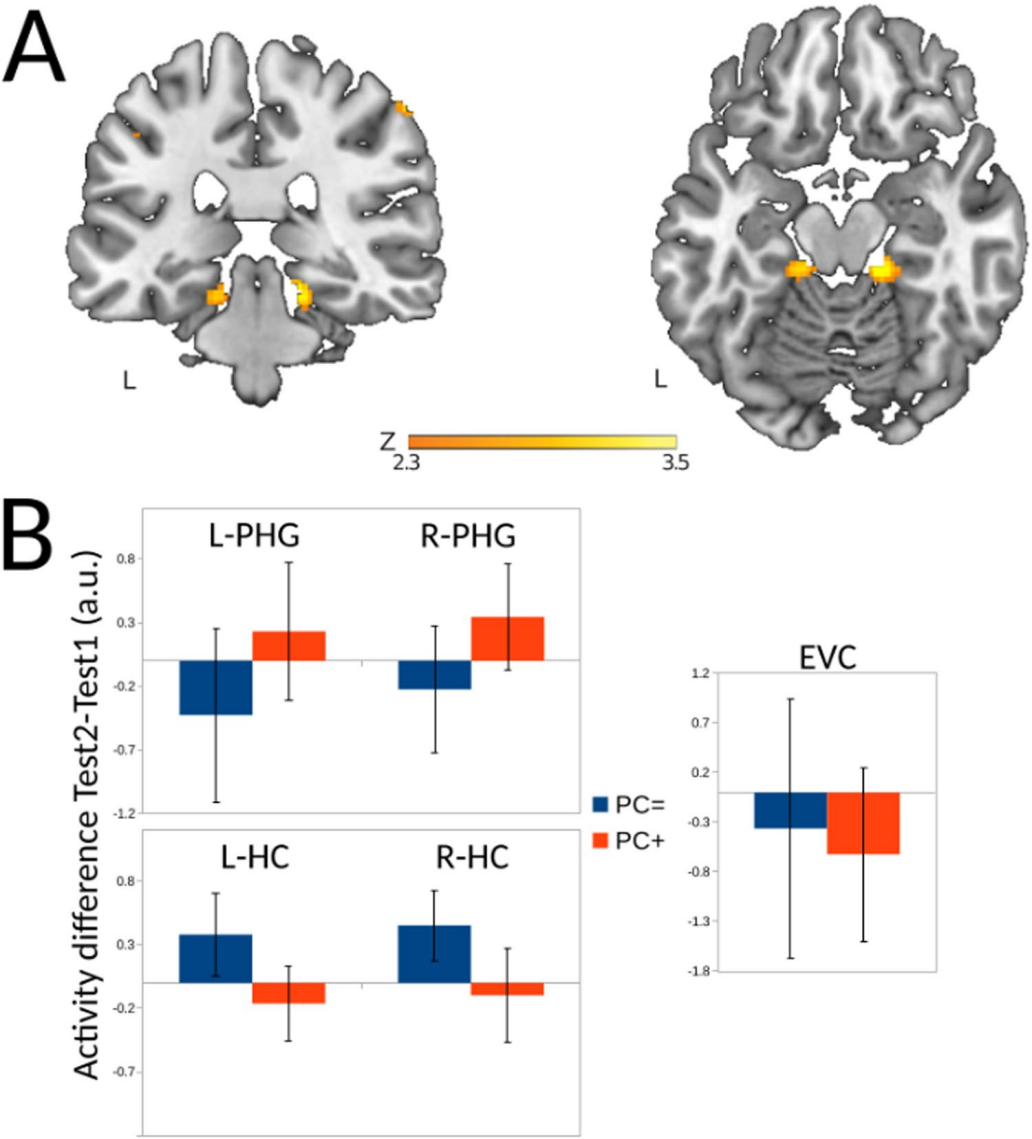
Overnight activity changes related to network integration of occipital cortex (**A**) Regions in the parahippocampal gyrus where there is a relation, on the one hand, between an activity increase between the pre-sleep and post-sleep test and, on the other hand, increased network integration of occipital cortex during cueing in the between Test1 and Test 2. Parametric maps were superimposed onto a template brain, using the cluster-forming threshold of Z > 2.33. (**B**) For comparison purposes, the mean overnight regional activation differences (calculated as activity during post-sleep Test 2 minus pre-sleep Test 1) are plotted for the left (L-PHG) and right (R-PHG) parahippocampal gyrus, the left (L-HC) and right (R-HC) hippocampus, and early visual cortex (EVC) for two groups of participants based on a median split on the scores of the participation coefficient in the early visual cortex. Even though there were no significant differences here, the numerical values are broadly in agreement with our hypotheses, namely finding a reduced activity of the hippocampus at Test 2 and an increased activity of the parahippocampal gyrus for the PC+ group (those participants that did display the strongest increase of the participation coefficient) versus the PC = group (those participants that displayed the lowest increase of the participation coefficient)and no visible differences in the early visual cortex.

We then considered whether the increased network integration of the occipital cortex induced by cueing during sleep had subsequent effects on neural activity when retrieving object-locations during the post-sleep test. Here, we found that the participation coefficient was related to a higher retrieval-related activity in Test 2 relative to Test 1 in both left parahippocampal gyrus (peak MNI coordinates: *x, y, z* = [−16, −32, −18], *Z*_21_ = 4.14), and right parahippocampal gyrus (peak MNI coordinates: *x, y, z* = [16, −32, −18], *Z*_21_ = 3.26, cluster-forming threshold at >2.33, corrected for a reduced search volume based on anatomical masks of left and right parahippocampal gyrus at a threshold of *p* < 0.05 using Gaussian Random Field Theory). Thus, increased network integration of occipital cortex during cueing in sleep had a subsequent effect on neural regions activated during retrieval of object-locations at Test 2, thereby recruiting bilateral parahippocampal gyrus at Test 2 to a relatively greater extent (see Fig. 4 and Table 3).

**Table 3:** Brain regions that show a significant relation between an increase in network integration of occipital cortex during cueing in slow-wave sleep and activity on the post-sleep test versus the pre-sleep test.

#	Region	X	Y	Z	Peak Z-value	Cluster Size	P-value
1	L parahippocampal gyrus	−16	−32	−18	3.26	40	0.038*
2	R parahippocampal gyrus	16	−32	−18	2.81	215	0.013*

Listed are clusters found in pre-defined anatomical regions of interest. The critical p-value was calculated to correct for multiple (2) comparisons using anatomical regions of interest using the Benjamin & Hochberg procedure (1995) to control the false-discovery rate. The critical p-value thus calculated was p = 0.038.

*Region was significant using a multiple comparison correction for a reduced search volume defined by anatomical regions of interest, and a subsequent correction for the false discovery rate (critical p-value = 0.038).

## Discussion

Here, we investigated the whole-brain neural network reorganization induced by cued reactivation of memory traces during deep sleep, and its relation to behavioral and neural responses during subsequent retrieval of those memories. For that purpose, we re-analyzed fMRI data reported in the study by van Dongen and colleagues^13^ using whole-brain connectivity measures. We demonstrated that when participants were in a state of slow-wave sleep, absent of visual input, and were cued with sounds associated with previously learned visuospatial information, the occipital cortex displayed increased network integration as measured with the participation coefficient. Furthermore, subjects who displayed increased cue-induced network integration of occipital cortex showed increased memory stabilization. This finding is congruent with the notion that the sound cue induces global reprocessing of the learning episode that may be conducive to subsequent memory retention. Although occipital cortex is not typically a focal point for mnemonic processing, previous studies have shown that cue-triggered reactivation can lead to instantiations of previously experienced episodes in this region^43,44^. It is likely that this reinstatement is coordinated with other mnemonic regions. Indeed, various regions displayed increased connectivity with occipital cortex during the presentation of cue sounds: memory and replay-related regions, such as the hippocampus, thalamus and medial prefrontal cortex, and higher-order associative cortices implicated in the encoding of the memory trace, such as the parahippocampal gyrus. These results are in line with a study^4^ that showed coordinated replay between the hippocampus and occipital cortex, the perceptual region that was involved in the initial processing of the stimulus. Cueing during slow-wave sleep also showed an enduring relation to neural activation at memory retrieval as measured during the post-sleep retrieval test. Specifically, subjects with greater network integration of occipital cortex during slow-wave sleep recruited parahippocampal gyrus more at subsequent retrieval relative to pre-sleep retrieval.

First, it should be reiterated that auditory cueing did not have an overall effect on memory retention of cued versus uncued associations. Similarly, the network reorganization found during the presentation of cue sounds was not specifically related to memory stabilization of those object-location memories associated with the cues. The association of network participation with memory stabilization was numerically larger for cued versus uncued material, but the interaction was not statistically significant. However, there could be various explanations for a nonspecific effect across all associations. First, the MRI scanning environment is extremely loud, unlike the quiet environment that would be preferred for auditory stimulation during sleep. Subjects who succeeded in falling asleep were also the ones who succeeded in filtering out all sensory input. Therefore, it is conceivable that the acoustic stimuli effectively modulated ongoing brain processes in some individuals but not others, contributing to large inter-individual differences and a lower power of demonstrating this effect as a group than in studies conducted outside of the MR-scanner environment^11^. It is also possible that the study was underpowered to observe an interaction effect, and that a larger sample would have rendered a significant interaction of cueing and network participation of the early visual cortex on overnight memory change. Furthermore, it is possible that the cueing had a non-specific effect on all material acquired within the same temporal and spatial learning context (as also suggested by the results reported in^9,12^). Indeed, all object-location associations were acquired within the same learning context; the same scanning session and temporal learning sequence, and most importantly, all objects were located on the same two-dimensional spatial grid. Therefore, it is plausible that the various object-locations became integrated into a unitary map, or spatial schema^45,46^ at initial learning and/or during replay^47,48^. As such, object locations could have been stored not only with reference to the two-dimensional grid, but also directly or indirectly in spatial reference to the other objects. Consequently, a sound cue could have reactivated cue-sound specific object-location associations, but also objects located within the objects’ vicinity. In fact, it has been proposed that sleep-dependent memory replay is instrumental in the integration of memories into a cognitive schema^49^. Thus, while network integration of the occipital cortex during cueing is demonstrated to be positively related to overall memory stabilization of a set of object-location associations, the fact that an integrated spatial schema was reactivated could have contributed to non-specific reactivation of all object-locations within this set. Previous studies might have been powerful enough to find a cue-selective effect of targeted memory reactivation^10,11^, over and above this context-general effect. Future studies using targeted memory reactivation should control for this by testing uncued material learned in the same context as the cued material, along with uncued material acquired in a different learning context, in order to disentangle these effects.

An alternative explanation of the brain-behavior relationship can be proffered too. It could be that for those subjects who display better memory consolidation across sleep, re-exposure of the cues during sleep induces a better memory reactivation. Here, a stronger association strength between the cue and the memory trace during slow-wave sleep prior to cueing could result in stronger memory reactivation following cue presentation. This alternative explanation, where reactivation does not improve memory but rather is a result of better memory, cannot be ruled out based on the current data.

Bearing in mind these caveats, our results show a network reorganization in response to cues presented during slow-wave sleep that is consistent with an active model of sleep-dependent memory consolidation. Previous research has shown that whole-brain connectivity patterns display a general reduction in thalamo-cortical and cortico-cortical connectivity and an increase in local clustering during slow-wave sleep^50^. Against this backdrop, a hippocampal-neocortical dialogue takes place facilitating systems-level consolidation by integrating hippocampal-dependent memories into neocortical storage sites^51,52^. This hippocampal-neocortical dialogue is orchestrated through oscillatory electrophysiological patterns characteristic of and related to memory replay^53,54^. Specifically, slow (delta wave) oscillations propagate across the brain and to the medial temporal lobes, including the hippocampus, exerting a global control over spiking activity^55,56^. Mechanistically, the up-state of slow oscillations has been found to enable thalamic sleep spindles in the sigma band, which in turn cluster high-frequency hippocampal ripples in their troughs. These hippocampal ripples then are proposed to allow for a precisely timed exchange of mnemonic information between the hippocampus and neocortex, potentiating and strengthening the neocortical memory trace.

If external auditory cueing would induce a reactivation of the visuo-spatial memory traces, one would expect that cortical representation areas corresponding to the modality of the memory trace would be recruited to participate in this cross-talk. Indeed, the occipital cortex displayed an increase in global network integration during cueing, and when probing the neural connectivity changes paired with this increase, we found that this region increases its connectivity with the regions involved in active cross-talk during slow-wave sleep, namely the hippocampus, thalamus and medial prefrontal cortex^17,57^. Of note, our findings do not exclude the possibility that connectivity within the visual cortex is also increased in response to cueing. The findings merely indicate that inter-modular connectivity is preferentially increased. A reinstated memory trace consists of connections between lower-level perceptual regions such as the early visual cortex (representing low-level features) and higher-level associative/mnemonic regions such as the hippocampus and parahippocampal gyrus (which bind together low-level features^58,59^). Moreover, memory reactivation during sleep is thought to be paired with a synchronization of neural activity across disparate brain regions, facilitating the exchange of information proposed to be necessary for systems-level memory consolidation^16,17,26^. Therefore, an increase in inter-modular connectivity of regions representing features of the memory trace reflects processes expected to be induced by memory cueing.

It is plausible that the neural results reported here are akin to those found during endogenous memory replay predominantly occurring during slow-wave sleep. Certainly, the neural patterns reported here match presumed neural mechanisms of memory replay during slow-wave sleep. However, the same neural pattern may also be found in response to memory reactivation during other brain states, such as wake, light sleep or REM-sleep. Based on the current data, the extent to which the neural memory reactivation pattern described is specific to slow-wave sleep cannot be assessed, as cues were only presented during slow-wave sleep. Future studies may compare the neural effects of cued memory reactivation across different states to determine which aspects are specific to slow-wave sleep and which are state-independent.

At first sight, it might be surprising that the auditory cortex was not recruited in response to auditory cueing. However, it should be noted that a contrast was made between two conditions in which sounds were presented. The critical difference in the contrast was that cue sounds but not control sounds were associated with specific visuo-spatial memories. Given the multimodal learning conditions, one could still expect increased connectivity of the auditory cortex with visual, spatial, and memory regions during cueing. As the functional connectivity analyses were informed by results of the graph theory analyses, we did not further probe connectivity from an anatomically defined seed in auditory cortex. Despite this caveat, it remains to be noted that cueing recruited occipital cortex to engage with mnemonic regions that have been shown to display increased cross-talk during slow-wave sleep, and this was related to the stabilization of the associated memory traces in neocortical storage sites.

Sleep supports memory retention in a selective manner, and information is prioritized based on perceived future relevance at encoding^60,61^ emotional salience^62–64^ and consistency across episodes and with prior knowledge^47,65^. Possibly, relevant information is selectively prioritized for active processing during sleep, whereas non-prioritized memory traces are forgotten, perhaps aided by homeostatic regulation of synaptic plasticity through global downscaling^66–68^. The net result is an increase in signal-to-noise for the prioritized memory traces in the neocortex, contributing to memory stabilization. Indeed, this appears to be the case in the parahippocampal gyrus, where an increase in retrieval-related activation of posterior parahippocampal gyrus at post-sleep test versus pre-sleep test was related to an increase in network participation of occipital cortex during cueing in sleep. Parahippocampal gyrus is known to be involved in representing the local visual environment^33^ and learning spatial layouts^31^. The increased involvement of parahippocampal gyrus during post-sleep retrieval is consistent with a role of parahippocampal gyrus in binding together low-level visual information from downstream occipital cortex. Notably, parahippocampal gyrus was activated during retrieval at the pre-sleep test, showed selective increased activation in response to cueing, and displayed increased connectivity with occipital cortex during cued reactivation, as originally reported^13^. The results reported here further elucidate a potential mechanism involved in sleep-based memory reactivation by taking a network perspective.

In sum, here we show that inducing the reactivation of object-location memories with associated auditory cues during slow-wave sleep increases the integration of information in the occipital cortex within a global brain network, specifically including those regions involved in memory replay, such as the medial temporal lobe, thalamus and medial prefrontal cortex. Furthermore, global network integration of the occipital cortex during cueing showed a correlation with overnight memory stabilization of learned materials and involvement of the parahippocampal gyrus during post-sleep retrieval. These findings highlight how graph theory analysis can be used to assess whole-brain connectivity patterns during targeted memory reactivation in slow-wave sleep, and contribute to a better understanding of sleep-related memory consolidation.

## Electronic supplementary material

Supplementary Figure 1

## Data Availability

The datasets generated during and/or analyzed during the current study are available from the corresponding author on reasonable request.

## References

[1] Diekelmann S, Büchel C, Born J, Rasch B (2011). Labile or stable: opposing consequences for memory when reactivated during waking and sleep. Nat. Neurosci..

[2] Diekelmann S, Born J (2010). The memory function of sleep. Nat. Rev. Neurosci..

[3] Wilson MA, McNaughton BL (1994). Reactivation of hippocampal ensemble memories during sleep. Science.

[4] Ji D, Wilson MA (2007). Coordinated memory replay in the visual cortex and hippocampus during sleep. Nat. Neurosci..

[5] Euston DR, Tatsuno M, McNaughton BL (2007). Fast-forward playback of recent memory sequences in prefrontal cortex during sleep. Science.

[6] Peyrache A, Khamassi M, Benchenane K, Wiener SI, Battaglia FP (2009). Replay of rule-learning related neural patterns in the prefrontal cortex during sleep. Nat. Neurosci..

[7] Genzel, L. & Battaglia, F. P. Cortico-Hippocampal Circuits for Memory Consolidation: The Role of the Prefrontal Cortex. In *Cognitive Neuroscience of Memory Consolidation* 265–281 (Springer, 2017).

[8] Dupret D, O’Neill J, Pleydell-Bouverie B, Csicsvari J (2010). The reorganization and reactivation of hippocampal maps predict spatial memory performance. Nat. Neurosci..

[9] Rasch B, Büchel C, Gais S, Born J (2007). Odor cues during slow-wave sleep prompt declarative memory consolidation. Science (80-.)..

[10] Creery JD, Oudiette D, Antony JW, Paller KA (2015). Targeted memory reactivation during sleep depends on prior learning. Sleep.

[11] Rudoy JD, Voss JL, Westerberg CE, Paller KA (2009). Strengthening individual memories by reactivating them during sleep. Science.

[12] Oudiette D, Antony JW, Creery JD, Paller KA (2013). The role of memory reactivation during wakefulness and sleep in determining which memories endure. J. Neurosci..

[13] van Dongen EV (2012). Memory stabilization with targeted reactivation during human slow-wave sleep. Proc. Natl. Acad. Sci..

[14] Ong JL (2016). Effects of phase-locked acoustic stimulation during a nap on EEG spectra and declarative memory consolidation. Sleep Med..

[15] Schouten DI, Pereira SIR, Tops M, Louzada FM (2017). State of the art on targeted memory reactivation: sleep your way to enhanced cognition. Sleep Med. Rev..

[16] Bergmann TO, Mölle M, Diedrichs J, Born J, Siebner HR (2012). Sleep spindle-related reactivation of category-specific cortical regions after learning face-scene associations. Neuroimage.

[17] Staresina, B. P. *et al*. Hierarchical nesting of slow oscillations, spindles and ripples in the human hippocampus during sleep. *Nat. Neurosci* (2015). 10.1038/nn.4119 PMC4625581 26389842

[18] Bullmore E, Sporns O (2009). Complex brain networks: graph theoretical analysis of structural and functional systems. Nat. Rev. Neurosci..

[19] Power JD, Schlaggar BL, Lessov-Schlaggar CN, Petersen SE (2013). Evidence for hubs in human functional brain networks. Neuron.

[20] DeYoe EA (1996). Mapping striate and extrastriate visual areas in human cerebral cortex. Proc. Natl. Acad. Sci..

[21] Aminoff E, Gronau N, Bar M (2007). The parahippocampal cortex mediates spatial and nonspatial associations. Cereb. Cortex.

[22] American Academy of Sleep Medicine. *The AASM manual for the scoring of sleep and associated events: Rules, Terminology and Technical Specifications*. AASM (2007).

[23] Schmitter S (2008). Silent echo-planar imaging for auditory FMRI. Magn. Reson. Mater. Physics, Biol. Med..

[24] Ekman M, Derrfuss J, Tittgemeyer M, Fiebach CJ (2012). Predicting errors from reconfiguration patterns in human brain networks. Proc. Natl. Acad. Sci..

[25] Rissman J, Gazzaley A, D’Esposito M (2004). Measuring functional connectivity during distinct stages of a cognitive task. Neuroimage.

[26] Marshall L, Born J (2007). The contribution of sleep to hippocampus-dependent memory consolidation. Trends Cogn. Sci..

[27] Guimera R, Mossa S, Turtschi A, Amaral LAN (2005). The worldwide air transportation network: Anomalous centrality, community structure, and cities’ global roles. Proc. Natl. Acad. Sci..

[28] Backus Alexander R., Bosch Sander E., Ekman Matthias, Grabovetsky Alejandro Vicente, Doeller Christian F. (2016). Mnemonic convergence in the human hippocampus. Nature Communications.

[29] Rubinov M, Sporns O (2010). Complex network measures of brain connectivity: uses and interpretations. Neuroimage.

[30] Tzourio-Mazoyer N (2002). Automated anatomical labeling of activations in SPM using a macroscopic anatomical parcellation of the MNI MRI single-subject brain. Neuroimage.

[31] Blondel VD, Guillaume J-L, Lambiotte R, Lefebvre E (2008). Fast unfolding of communities in large networks. J. Stat. Mech. theory Exp..

[32] Aguirre GK, Detre JA, Alsop DC, D’Esposito M (1996). The parahippocampus subserves topographical learning in man. Cereb. cortex.

[33] Epstein R, Kanwisher N (1998). A cortical representation of the local visual environment. Nature.

[34] Epstein R, Harris A, Stanley D, Kanwisher N (1999). The parahippocampal place area: recognition, navigation, or encoding?. Neuron.

[35] Danker JF, Anderson JR (2010). The ghosts of brain states past: remembering reactivates the brain regions engaged during encoding. Psychol. Bull..

[36] Nyberg L, Habib R, McIntosh AR, Tulving E (2000). Reactivation of encoding-related brain activity during memory retrieval. Proc. Natl. Acad. Sci..

[37] Polyn SM, Natu VS, Cohen JD, Norman KA (2005). Category-specific cortical activity precedes retrieval during memory search. Science.

[38] Mölle M, Marshall L, Gais S, Born J (2002). Grouping of spindle activity during slow oscillations in human non-rapid eye movement sleep. J. Neurosci..

[39] Skaggs WE, McNaughton BL (1996). Replay of neuronal firing sequences in rat hippocampus during sleep following spatial experience. Science.

[40] Aleman-Gomez, Y., Melie-Garcia, L. & Valdes-Hernandez, P. A. IBASPM: Toolbox for automatic parcellation of brain structures. In *12th Annual Meeting of the Organization forHuman Brain Mapping. June* 11-15, 2006. *Florence, Italy* (2006).

[41] Benjamin, Y. & Hochberg, Y. Controlling the false discovery rate: a practical and powerful approach to multiple testing. *J. R. Stat. Soc. Ser. B* 289–300 (1995).

[42] Peigneux P (2004). Are spatial memories strengthened in the human hippocampus during slow wave sleep?. Neuron.

[43] Xu S, Jiang W, Poo M, Dan Y (2012). Activity recall in a visual cortical ensemble. Nat. Neurosci..

[44] Ekman Matthias, Kok Peter, de Lange Floris P. (2017). Time-compressed preplay of anticipated events in human primary visual cortex. Nature Communications.

[45] Tse D (2007). Schemas and Memory Consolidation. Sci..

[46] van Buuren M (2014). Initial investigation of the effects of an experimentally learned schema on spatial associative memory in humans. J. Neurosci..

[47] Durrant SJ, Cairney SA, McDermott C, Lewis PA (2015). Schema-conformant memories are preferentially consolidated during REM sleep. Neurobiol. Learn. Mem..

[48] Hennies N, Ralph MAL, Kempkes M, Cousins JN, Lewis PA (2016). Sleep spindle density predicts the effect of prior knowledge on memory consolidation. J. Neurosci..

[49] Lewis PA, Durrant SJ (2011). Overlapping memory replay during sleep builds cognitive schemata. Trends Cogn. Sci..

[50] Spoormaker VI (2010). Development of a large-scale functional brain network during human non-rapid eye movement sleep. J. Neurosci..

[51] Buzsáki G (1996). The hippocampo-neocortical dialogue. Cereb. cortex.

[52] Mitra Anish, Snyder Abraham Z., Hacker Carl D., Pahwa Mrinal, Tagliazucchi Enzo, Laufs Helmut, Leuthardt Eric C., Raichle Marcus E. (2016). Human cortical–hippocampal dialogue in wake and slow-wave sleep. Proceedings of the National Academy of Sciences.

[53] Diba K, Buzsáki G (2007). Forward and reverse hippocampal place-cell sequences during ripples. Nat. Neurosci..

[54] Davidson TJ, Kloosterman F, Wilson MA (2009). Hippocampal replay of extended experience. Neuron.

[55] Massimini M, Huber R, Ferrarelli F, Hill S, Tononi G (2004). The sleep slow oscillation as a traveling wave. J. Neurosci..

[56] Nir Y (2011). Regional slow waves and spindles in human sleep. Neuron.

[57] Mölle M, Yeshenko O, Marshall L, Sara SJ, Born J (2006). Hippocampal sharp wave-ripples linked to slow oscillations in rat slow-wave sleep. J. Neurophysiol..

[58] Alvarez P, Squire LR (1994). Memory consolidation and the medial temporal lobe: a simple network model. Proc. Natl. Acad. Sci..

[59] Nadel L, Samsonovich A, Ryan L, Moscovitch M (2000). Multiple trace theory of human memory: computational, neuroimaging, and neuropsychological results. Hippocampus.

[60] van Dongen EV, Thielen J-W, Takashima A, Barth M, Fernández G (2012). Sleep supports selective retention of associative memories based on relevance for future utilization. PLoS One.

[61] Wilhelm I (2011). Sleep selectively enhances memory expected to be of future relevance. J. Neurosci..

[62] Hu P, Stylos-Allan M, Walker MP (2006). Sleep facilitates consolidation of emotional declarative memory. Psychol. Sci..

[63] Wagner U, Hallschmid M, Rasch B, Born J (2006). Brief sleep after learning keeps emotional memories alive for years. Biol. Psychiatry.

[64] Payne JD (2007). Stress administered prior to encoding impairs neutral but enhances emotional long-term episodic memories. Learn. Mem..

[65] Tamminen J, Ralph MAL, Lewis PA (2013). The role of sleep spindles and slow-wave activity in integrating new information in semantic memory. J. Neurosci..

[66] Tononi G, Cirelli C (2003). Sleep and synaptic homeostasis: a hypothesis. Brain Res. Bull..

[67] Tononi G, Cirelli C (2006). Sleep function and synaptic homeostasis. Sleep Med. Rev..

[68] Genzel L, Kroes MCW, Dresler M, Battaglia FP (2014). Light sleep versus slow wave sleep in memory consolidation: a question of global versus local processes?. Trends Neurosci..

