# Supplementary material for: Cued reactivation during slow-wave sleep induces brain connectivity changes related to memory stabilization
Source: Sci Rep. 2018 Nov 16;8:16958. doi: 10.1038/s41598-018-35287-6 (PMC6240046; doi:10.1038/s41598-018-35287-6)
Supplement: Supplementary file 1 — Supplementary Figure 1 [file 41598_2018_35287_MOESM1_ESM.doc]

Supplementary Information

Accompanying the manuscript:

“Cued reactivation during slow-wave sleep induces brain connectivity changes related to memory stabilization.”

By: Ruud M.W.J. Berkers, Matthias Ekman, Eelco V. van Dongen, Atsuko Takashima, Markus Barth, Ken A. Paller, Guillén Fernández.

Supplementary Figure

**
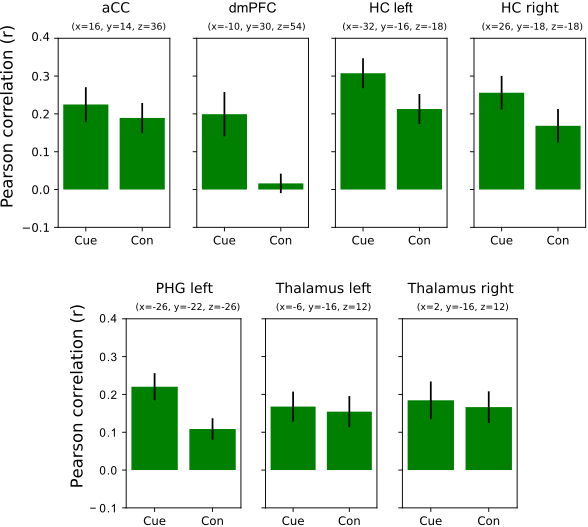
**

**Supplementary Figure 1**.

**Coupling with occipital cortex during cueing in SWS .** Correlation values for cue sounds and control sounds extracted for peaks of reported clusters. *aCC*, anterior Cingulate Cortex; *dmPFC*, dorsomedial prefrontal cortex, *HC*, Hippocampus, *PHG,* Parahippocampal Gyrus. Error bars depict s.e.m.
